# Prosthetic Valve Endocarditis: A Retrospective Cohort Study Conducted at “Dr. Carol Davila” Central Military Emergency University Hospital in Bucharest

**DOI:** 10.3390/microorganisms12071442

**Published:** 2024-07-16

**Authors:** Corina-Ioana Anton, Cosmin Alexandru Buzilă, Silviu Marcel Stanciu, Săndica Bucurică, Daniela Anghel, Alexia Teodora Ștefan, Ion Ștefan, Adrian Streinu-Cercel

**Affiliations:** 1 Department of Infectious Diseases, “Dr. Carol Davila” Central Military Emergency University Hospital, 134 Calea Plevnei, 010242 Bucharest, Romania; corina-ioana.anton@drd.umfcd.ro; 2 Department of Medico-Surgical and Prophylactic Disciplines, Titu Maiorescu University, 040441 Bucharest, Romania; 3 Faculty of General Medicine, Carol Davila University of Medicine and Pharmacy, 8 Eroii Sanitari Bvd, 050474 Bucharest, Romania; 4 Cardiovascular Surgery Department, “Dr. Carol Davila” Central Military Emergency University Hospital, 134 Calea Plevnei, 010242 Bucharest, Romania; 5 Center for Cardiovascular Diseases, Laboratory of Noninvasive Cardiovascular Functional Explorations, Dr. Carol Davila Central Military Emergency University Hospital, 134 Calea Plevnei Str., 010825 Bucharest, Romania; 6 Department of Gastroenterology, “Dr. Carol Davila” Central Military Emergency University Hospital, 134 Calea Plevnei, 010242 Bucharest, Romania; 7 Department of Gastroenterology, Carol Davila University of Medicine and Pharmacy, 020021 Bucharest, Romania; 8 Department of Internal Medicine, “Dr. Carol Davila” Central Military Emergency University Hospital, 010242 Bucharest, Romania; 9 Department of Infectious Diseases I, Faculty of Medicine, Carol Davila University of Medicine and Pharmacy, 020021 Bucharest, Romania; 10 National Institute for Infectious Diseases “Prof. Dr. Matei Balş”, 1 Dr. Calistrat Grozovici Street, 021105 Bucharest, Romania

**Keywords:** prosthetic valve endocarditis, etiology, multidisciplinary treatment, secondary diagnosis, mortality rate

## Abstract

Objective: To evaluate patients with prosthetic valves who developed infective endocarditis by comparing treatment outcomes in both early- and late-onset IE episodes following prosthetic valve replacement surgery. This study sought to conduct a comprehensive assessment of the efficacy of these methodologies. The insights derived from this assessment can be utilized to enhance the quality of care for individuals with infective endocarditis who have undergone prosthetic valve replacement surgery. Results: During the period of investigation (January 2017–December 2022), 78 patients diagnosed with infective endocarditis (IE) on a prosthetic valve were admitted to the Infectious Diseases Department of the “Dr. Carol Davila” Central Military Emergency University Hospital in Bucharest. In 28 patients (35.8%), the onset of PVE occurred within 12 months of surgery (early onset), whereas in 50 patients (64.2%), the onset occurred more than 12 months after surgery (late onset). The mortality rate was 35.9% (53.6% among the early onset patients and 26% among the late-onset patients). Among patients who received surgical and medical therapy, the mortality rate was 29.6%, whereas among those who received only medical therapy, a 39.2% mortality rate was reported. According to the extracted data, antibiotic therapy was successful in 72.6% of the patients. In contrast, a combination of surgical and drug-based approaches resulted in a cure in 76.1% of patients. The most common etiological agent was *Staphylococcus aureus* (38.5%), followed by *Enterococcus faecalis* (26.9%) and *Streptococcus mitis* (10.3%). The mortality rate of patients infected with *S. aureus* was 29.2%, indicating the severity of this infectious agent. Conclusions: Prosthetic valve endocarditis (PVE) is a serious condition associated with a high mortality rate both in the short and long term. Regardless of the therapy used, the risk of death remains high.

## 1. Introduction

Infective endocarditis (IE) is a condition that has not seen a substantial reduction in mortality rates despite the progress made in current developments in antibiotic discovery, the use of advanced imaging techniques such as transesophageal echocardiography for early diagnosis, and the implementation of treatment efforts in recent years [1].

IE, while still relatively rare, appears to be on the rise. This severe form of sepsis affects up to 40–50% of patients, who may require valve surgery. With a mortality rate of 20–25% per year, the disease remains a significant concern. However, the classic clinical syndromes of acute or subacute endocarditis have shifted, and the current forms are less pronounced [2,3].

Notably, high-income countries have experienced profound epidemiological changes, with a growing number of cases involving prosthetic valves.

Prosthetic valve infective endocarditis (PVE) was initially identified in the 1950s and has been the focus of numerous studies reporting extremely high mortality rates among these patients. PVE is a serious condition that can arise following cardiac valve replacement surgery, and it is associated with elevated mortality rates, which can range from 18% to 59% [4,5].

PVE is a severe and potentially life-threatening complication of valve replacement surgery, with an incidence of 10–30% of all IE cases. Patients with prosthetic heart valves are considered to be at high risk of developing IE, with an incidence rate of 0.3–1.2%. Approximately one-third of all endocarditis cases are attributed to PVE, due to the growing number of valve replacement procedures being performed. The rise in transcatheter aortic valve implantations (TAVIs) has further contributed to the prevalence of this condition [6,7].

Research suggests that the mitral and aortic valves are most commonly affected, while the tricuspid valve is least commonly affected [7,8,9].

Bacteria are the primary cause of prosthetic valve endocarditis (PVE), although fungal species can also lead to IE [9,10].

*S. aureus*, *E. faecalis*, and *Coagulase-negative Staphylococci* are the most commonly implicated bacteria in the pathogenesis of IE [10].

Surgical management of patients diagnosed with PVE typically targets cases in which significant vegetation, prosthetic valve infections, valve dysfunction, or advanced cardiac insufficiency are identified through sonography. Frequently, when antibiotics fail to alleviate septic symptoms, such as fever, chills, fatigue, and low blood pressure that persist for over a week, surgical intervention involving a prosthetic valve is usually warranted [11,12].

The outcome of PVE is often determined by the time of presentation. Early-onset PVE occurs less than one year after surgery, while late-onset takes place at more than a year after surgical treatment.

Patients diagnosed with PVE may experience a variety of complications, including persistently positive blood cultures, septic shock, or even death. It is crucial to understand that persistent infection and heart failure are the most significant predictive factors of mortality in hospitalized patients with PVE. Complications associated with PVE are often challenging to manage, and the complexity of the condition typically requires specialized knowledge and expertise to effectively address the associated issues [12,13,14].

*S. aureus* is still associated with high mortality rates in patients with PVE, as well as early complications such as congestive heart failure. The postoperative mortality rates in this category remained high, indicating virulence of the infectious pathogen. The prognosis for these individuals is challenging to evaluate because it can fluctuate significantly depending on the specific infectious agent present [15,16].

Two important innovations in the management of PVE have been suggested in recent years: a multimodal approach and a team of experts that constitutes the endocarditis team [13].

Several studies support the importance of multidisciplinary therapy for IE (infectious disease specialists, cardiac surgeons, cardiologists, neurologists, and anesthesiologists) that are bound to guide medical therapy [12,13,14].

The primary responsibility of the endocarditis team is to evaluate the most vulnerable or severely impaired patients, as there is still considerable debate regarding the optimal timing and postoperative care for these patients. The decision to perform surgery must consider various factors, including the location and severity of the infection, the patient’s preoperative condition and comorbidities, and the feasibility of early surgical intervention. The treatment of PVE is among the most difficult, often resulting in a high mortality rate [14,15,16].

Patients with PVE are more likely to experience complications and unfavorable outcomes than those with native valve endocarditis, despite having a similar incriminating agent. Thus, it is essential to diagnose PVE accurately and initiate prompt treatment to mitigate its detrimental effects. The significance of the multimodal approach for the diagnosis and management of PVE has become increasingly recognized [17,18].

## 2. Materials and Method

### 2.1. Study Design

This was an observational, population-based cohort study that utilized data from the electronic records of individuals who were admitted to the Infectious Diseases Department of the “Dr. Carol Davila” Central Military Emergency University Hospital in Bucharest between 1 January 2017 and 31 December 2022. The study was conducted in accordance with ethical guidelines and approved by the Ethics Committee of the “Dr. Carol Davila” Central Military Emergency University Hospital in Bucharest (Decision No. 562/20.12.2022). Informed consent was obtained from all the patients included in the study.

### 2.2. Setting

PVE was diagnosed according to the modified Duke criteria [7]. All patients who were diagnosed with PVE between 1 January 2017 and 31 December 2022 were included in the study. The follow-ups for PVE and survival ended on 31 December 2023 and February 2024, respectively.

### 2.3. Study Population

We included all patients (n = 78) who were diagnosed with PVE between 2017 and 2022 at the “Dr. Carol Davila” Central Military Emergency University Hospital in Bucharest. The exclusion criteria were other types of implantable cardiovascular devices, three consecutive negative blood cultures, and no obvious vegetation on transesophageal ultrasound examination. Three sets of blood cultures were obtained from each patient with a minimum interval of 30 min between each set and a maximum of 24 h. Blood cultures were collected, and patients were administered empirical broad-spectrum antibiotic therapy.

Following the receipt of blood culture results at an average interval of 3–5 days, antibiotic therapy was directed using an antibiogram. The decision to administer combined therapy, comprising medical treatment and surgery, was made by a multidisciplinary team that included an infectious disease specialist, cardiologist, and cardiovascular surgeon.

### 2.4. Statistical Analysis

Frequency tables and descriptive statistics (mean and standard deviation) were used to analyze patient information, and two non-parametric statistical tests, the Mann–Whitney test and Kruskal–Wallis test, were applied to reveal gender and age differences in patients’ medical characteristics. These non-parametric tests have the main advantage of not making any assumptions about the shape of the population distribution from which the sample was drawn. The results of the Kruskal–Wallis test are expressed as *p*-values. The *p*-value represents the probability of obtaining differences as large or larger than those observed in our data, if the null hypothesis is true. If the *p*-value is less than the predefined significance level of 0.05, we reject the null hypothesis and conclude that there are significant differences between at least two groups.

SPSS software Version 26 was used to conduct the statistical analyses.

### 2.5. Objective

The objective of this study was to evaluate patients with PVE by comparing the treatment outcomes of early- and late-onset IE episodes following prosthetic valve replacement surgery. The goal was to provide a comprehensive assessment of the efficacy of these methods. The findings from this assessment will be used to improve the quality of care for individuals who have undergone prosthetic valve replacement surgery for IE.

## 3. Results 

This study included a total of 78 patients. The patient distribution per year was as follows: 23 in 2017, 20 in 2018, 10 in 2019, 13 in 2020, and 12 in 2021. With regard to age, the majority of patients fell within the range of 61 to 80 years (74.4%). Of the patients, 14.2% were aged between 41 and 60 years and 3.84% were aged between 21 and 40 years. Only 7.6% of patients were aged >80 years. The participants were aged between 25 and 95 years, with a mean age of 66 years.

Upon admission, fever was the most common symptom that was present in all patients. In terms of hospitalization, the majority of the patients (66.4%) required hospitalization for 31–43 days, while 33.6% required more than 46 days.

The shortest hospital stay was 3 days, whereas the longest was 94 days, with an average hospital stay of 34.8 days. Evolutionarily, 27 patients (34.7%) were admitted to the ICU with an average duration of 11 days. For patients who were only treated with antibiotics, the length of hospital stay varied between a minimum of 5 days and a maximum of 94 days, with an average stay of 62.4 days. For patients who underwent surgical intervention in addition to antibiotic treatment, the minimum duration of hospitalization was 3 days, whereas the maximum duration was 73 days, with an average stay of 51.2 days.

### 3.1. Analysis of the Distribution of Etiological Agents in Patients with PVE

Microorganisms were detected in the blood cultures of all 78 patients using serological methods. The most prevalent microorganism identified was *S. aureus* (38.5%), followed by *E. faecalis* (26.9%). These data are shown in Table 1. 

Upon examining the dissemination of pathogenic organisms by age, it was found that the majority of patients in whom the primary causative factors were detected were in the 61–80 age cohort (Table 2).

The analysis of the gender distribution of the most prevalent infectious agent demonstrated that 36.1% of male patients and 41.9% of female patients had positive *S. aureus* blood cultures, as shown in Table 3.

### 3.2. Disease Onset

Examination of the onset of PVE indicated that 28 patients (35.8%) experienced early-onset PVE (within 12 months of surgery). Among these patients, 18 (64.2%) were male and 10 (35.3%) were female. 

Examination of the affected valves in patients with early-onset disease revealed that the aortic valve was affected in 21 patients (67.9%), of which 13 (84.2%) were male, and eight (15.8%) were female. The mitral valve was affected in seven patients (32.1%), of which five (55.6%) were male, and two (44.4%) were female.

**Figure 1:**
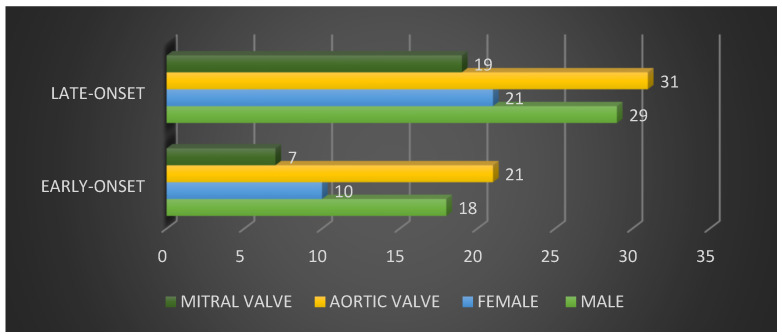
Distribution of early-onset and late-onset PVE according to gender and valve type.

Late-onset disease, which developed more than 12 months after surgery, represented 50 of the patients included in the study (64.2%), with gender distribution of 29 males (58%) and 21 females (42%). Examination of the affected valves in individuals with late-onset disease revealed that 31 patients (62%) had an affected aortic valve, of which 17 (54.8%) were male and 14 (42.2%) were female. Additionally, 19 patients (38%) had an affected mitral valve, of which 12 (63.2%) were male and seven (36.8%) were female. These data are shown in Figure 1.

The frequency of etiological agents according to the disease onset was also analyzed. The data are summarized in Table 4.

The distribution of infectious agents according to the time of onset and patient gender was analyzed. The results were summarized in Table 5.

The Mann–Whitney U test was used to assess whether any noticeable distinctions existed between patient groups infected with specific pathogens. For *S. aureus*, the *p*-value of 0.907 surpassed the conventional significance level of 0.05 (or 5%). However, for *E. faecalis*, the *p*-value of 0.054 fell below the standard significance level, thereby suggesting that there are notable discrepancies between patient groups affected by PVE caused by this pathogen.

### 3.3. Analysis of Secondary Diagnoses

Among the patients with PVE (n = 78), the majority had secondary diagnoses such as mitral valve insufficiency (36.3%), aortic valve insufficiency (31.2%), congestive heart failure (25.7%), and essential (primary) hypertension (29.4%).

The distribution of secondary diagnoses according to the age of the patients in the study revealed that the most affected age group was 61–80 years, followed by the age group 41–60 years.

Analysis of the distribution of the most prevalent secondary diagnoses for the age group of 61–80 years revealed that mitral insufficiency occurred in 73.2% of cases, aortic insufficiency in 65.2%, and congestive heart failure in 70.2%. Additionally, essential hypertension was associated with this age group in 78.1% of cases (Table 6).

Upon conducting an analysis of the prevalence of secondary diagnoses, the Kruskal–Wallis test indicated disparities among the various age groups with respect to cases of mitral valve insufficiency and aortic valve insufficiency. Specifically, the probability associated with the test was found to be below the conventional significance threshold of 8%. As a result, patients falling within the age range of 61 to 80 years are most probably diagnosed with secondary conditions such as mitral valve insufficiency and aortic valve insufficiency.

The distribution of secondary diagnoses based on patient gender was also analyzed. The results are summarized in Table 7.

### 3.4. Evaluation of Prosthetic Valve Pathology

**Figure 2:**
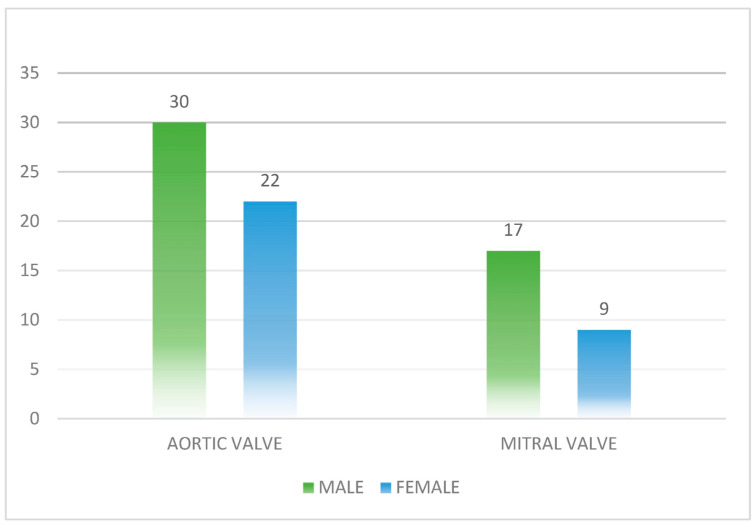
Distribution of prosthetic valve damage site by gender.

Based on the evaluation of prosthetic valve pathology, all 78 patients in this study had metallic prosthetic valves. Upon analyzing heart damage in these patients, it was found that the left heart was the most affected, with the aortic valve being affected in 52 patients (66.7%) and the mitral valve in 26 patients (33.3%). In terms of gender analysis of prosthetic valve damage, it was observed that in cases of aortic valve damage, 30 patients (57.7%) were male and 22 (42.3%) were female. For mitral valve damage, 17 patients (65.4%) were male and nine patients (34.6%) were female. These data are shown in Figure 2.

### 3.5. Treatment

Among the individuals included in this study, 51 (65.4%) received antibiotic treatment alone. Among them, 27 (52.9%) were male and 24 (47.1%) were female. In addition, 27 patients (34.6%) underwent a combination of medical and surgical interventions. Of these, 20 patients (74.1%) were male and seven patients (25.9%) were female. It is noteworthy that 74.1% of the surgical patients were aged 61–80 years.

Patients who underwent valve reoperation were older than those who received only medical therapy (27 patients, 60 ± 20 years vs. 45 ± 23 years; *p* < 0.001) and had a higher proportion of male patients (74.1% vs. 25.9%; *p* < 0.005).

In contrast, the incidence of *S. aureus* PVE was higher in patients who underwent surgery than in those who did not (41% vs. 35%; *p* = 0.002).

The duration of antibiotic treatment was notably shorter in the surgery group than that in the antibiotic group. Elective surgery was possible after 30 days of antibiotic treatment in 11 patients (40.7%). Emergency surgery was required in four patients (14.8%) due to unstable hemodynamic conditions, and among these patients, congestive heart failure occurred significantly more frequently than in those who underwent elective surgery.

The main reason for surgical intervention among the 20 patients (74.1%) was heart failure, which was followed by severe valvular insufficiency in seven patients (25.9%). It is noteworthy that 13 (48.1%) of these patients underwent surgery within 14 days of being hospitalized. The remaining patients were treated with alternative non-surgical options, which involved the administration of antibiotics.

All patients included in the studies received antibiotic therapy regardless of whether they were treated with medical or surgical therapy. The mean time from diagnosis to valve reoperation in the surgery group was 13 days.

Compared to medical therapy, valve reoperation outcomes were associated with a lower mortality rate.

### 3.6. Mortality Rate 

Upon analyzing the mortality rate of the patient population included in the study, 28 deaths (35.9%) were recorded. Of these, 17 (60.7%) were male and 11 (39.3%) were female.

In terms of mortality rate based on treatment, the following findings were observed: among patients receiving medical therapy only, 20 deaths (39.2%) were recorded. Of these, 12 (60%) were male, and 8 (40%) were female.

Among the patients who received both surgical and medical therapy, eight deaths (29.6%) were recorded. Of these, five patients (62.5%) were male and three (37.5%) were female.

In the surgery group, eight patients (29.6%) died during hospital admission, including one who died intraoperatively due to heart failure. Three patients died within the first 30 days after surgery due to multiorgan failure resulting from ongoing septicemia, acute myocardial infarction, or intracerebral hemorrhage. Another patient in the surgery group who had severe hypoxic brain damage from preoperative resuscitation died on day 80 due to pneumonia.

When analyzing the mortality rate according to the time of onset of IE episodes, the following were found: Among the 28 patients with early disease, 15 deaths (53.6%) were recorded. Of these, 11 patients (73.4%) were male and four patients (26.6%) were female. Among the 50 patients with late-onset disease, 13 died (26%). Of these, six patients (46.1%) were male and seven patients (53.9%) were female.

## 4. Discussions

The objective of this research was to investigate and assess the etiology, secondary diagnosis, valve pathology, and mortality rates correlated with different bacterial pathogens that lead to PVE in patients who received antibiotic treatment either alone or in conjunction with surgical therapy.

In the current investigation, PVE was diagnosed using the modified Duke criteria. The primary symptom that led to the suspicion of PVE was high fever, which was present in every case. This observation aligns with other studies that reported fever as the most common symptom [19,20].

Despite advancements in IE diagnosis and treatment of infective endocarditis, PVE remains a severe and potentially fatal complication. Consequently, a combination of medical therapy and surgery is typically regarded as the preferred course of action in managing this condition [5].

Numerous studies have demonstrated the superiority of surgical treatment over a strategy solely reliant on antibiotics. According to a more recent investigation, the medical group experienced a 46% mortality rate, whereas the surgical group reported a 26% mortality rate [5,20].

The present investigation, which evaluated the effectiveness of surgical intervention combined with antibiotic therapy versus medical management alone in patients with PVE, demonstrated that the combined approach was associated with a lower mortality rate than medical therapy. The findings of this study were consistent with those of previous studies [20,21].

In our study, we discovered that 35.8% of patients had early-onset PVE, while 64.2% had late-onset PVE. The prevalence of PVE in the aortic position was greater than that in the mitral position, at 67.9% compared with 32.1% in the early-onset group and 62% compared to 38% in the late-onset group, respectively. Our findings align with those of other studies that reported similar rates of PVE [21,22,23].

The primary etiological agent identified in our investigation was *S. aureus*, with *E. faecalis* following closely behind. In both the early- and late-onset groups, *S. aureus* was the most prevalent infectious agent, with *E. faecalis* being the second most common in the early-onset group and *S. mitis* in the late-onset group.

Notably, infection with *S. aureus* is frequently cited as the primary cause of PVE in numerous epidemiological studies and is considered a high-risk subgroup within this population [22].

The relationship between the infectious agent *S. aureus* and a deterioration of clinical outcomes in infective endocarditis (IE) has been established. Over the course of a 5-year study at our institution, *S. aureus* was the most frequently identified causative microorganism in patients with prosthetic valve endocarditis (PVE). As a pathogen known for its aggressive nature, *S. aureus* is often associated with a severe clinical presentation. These findings are consistent with those of previous studies [22,23].

A greater prevalence of PVE caused by *S. aureus* and *E. faecalis* has been documented. Studies have also revealed a more pronounced inclination of infection in mechanical valves. Mechanical valves are commonly associated with an increased susceptibility to PVE during the initial three months following surgery. Regardless of the time frame, *S. aureus* is typically identified as the primary pathogen associated with PVE. *S. aureus* has long been recognized as one of the most common bacterial pathogens associated with PVE, and its identification is crucial for effective treatment and prevention of the condition [23,24].

Our investigation revealed that the aortic valve was most frequently affected by PVE, comprising 66.7% of cases, while the mitral valve was affected in 33.3% of cases. These findings correspond with those of other studies on PVE [24,25,26].

In our study, the mortality rate was higher in the antibiotic group (39.3% vs. 29.6%), which is consistent with the majority of previous studies. Despite the high rates observed in the antibiotic group (39.2%), our findings on in-hospital mortality (35.9% mortality rate) were comparable to those published in major studies [27,28,29]

The risk of PVE in patients with prosthetic heart valves is high. The risk of infection was greater in the first 3 weeks after valve operation. In our study, we found that in the early-onset group, the mortality rate was higher, which is consistent with other studies that showed that most deaths occurred within 3 months after the onset of the disease [30].

Our results indicate that early intervention and prompt treatment are crucial for improving outcomes and reducing mortality rates in individuals with this condition. Therefore, it is essential to increase public awareness and accessibility to early intervention and prompt treatment for individuals with this condition [30,31].

The results of previous studies indicate that the decision to avoid surgery in individuals with surgical indications can have a considerable effect on their prognosis [31].

Notably, in the present study, patients who did not undergo surgery had a higher prevalence of comorbidities.

In PVE cases it has been shown that patients are older compared to those with IE on a native valve. As shown in previous studies, patients also have associated comorbidities [32].

The present investigation, which evaluated the effectiveness of surgical intervention combined with antibiotic therapy versus medical management alone in patients with PVE, demonstrated that the combined approach was associated with a lower mortality rate than medical therapy. The findings of this study were consistent with those of previous studies [33].

## 5. Conclusions

The present investigation indicated a lower mortality rate and greater survival at follow-up for individuals with PVE who underwent valve reoperations than for those who received medical therapy. Moreover, secondary diagnosis, infectious agents, and age also affected survival rates. PVE is a life-threatening illness that is often associated with a high mortality rate both in the short and long term. Despite the use of various therapies, the risk of death remains high. PVE is a complex ailment characterized by its diverse manifestations. Considering the growing utilization of valve prostheses, a proactive approach to prevention is essential. Shortening the duration between diagnosis and treatment is the most efficient strategy for minimizing mortality rates.

### Limitations

The current study had certain limitations that should be acknowledged. First, it was limited to only one hospital, which may have influenced the results. Future investigations should be conducted in a larger population to gain a more comprehensive understanding of the patient outcomes in PVE.

## Figures and Tables

**Table 1:** Distribution of etiologic agents from blood cultures among patients with PVE.

Etiologic Agent	Frequency	%
*Staphylococcus aureus*	30	38.5
*Enterococcus faecalis*	21	26.9
*Streptococcus mitis*	8	10.3
*Escherichia coli*	6	7.7
*Staphylococcus epidermidis*	5	6.3
*Streptococcus sanguinis*	4	5.1
*Streptococcus gallolyticus*	1	1.3
*Streptococcus pseudoporcinus*	1	1.3
*Enterobacter cloacae*	1	1.3
*Klebsiella pneumoniae*	1	1.3

**Table 2:** Distribution of infectious agents according to age.

	Age			
	21–40	41–60	61–80	81+
*Enterobacter cloacae*	0.0%	0.0%	1.1%	0.0%
*Enterococcus faecalis*	6.1%	18.3%	25.4%	11.6%
*Escherichia coli*	0.0%	5.7%	7.9%	4.1%
*Klebsiella pneumoniae*	0.0%	0.0%	0.0%	1.1%
*Staphylococcus aureus*	31.1%	28.6%	37.2%	31.5%
*Staphylococcus epidermidis*	0.0%	22.5%	65.4%	6.1%
*Streptococcus gallolyticus*	0.0%	0.0%	0.0%	1.1%
*Streptococcus mitis*	10.0%	66.6%	26,4%	0.0%
*Streptococcus pseudoporcinus*	0.0%	0.0%	1.1%	0.0%
*Streptococcus sanguinis*	21.0%	0.0%	0.0%	1.1%

**Table 3:** Distribution of infectious agents according to patient gender.

	Patient Gender	
	Male	Female
	%	%
*Enterobacter cloacae*	2.1%	0.0%
*Enterococcus faecalis*	**27.6%**	**25.8%**
*Escherichia coli*	2.1%	16.3%
*Klebsiella pneumoniae*	2.1%	0.0%
*Staphylococcus aureus*	**36.2%**	**41.9%**
*Staphylococcus epidermidis*	6.3%	6.4%
*Streptococcus gallolyticus*	2.1%	0.0%
*Streptococcus mitis*	**13.2%**	6.4%
*Streptococcus pseudoporcinus*	2.1%	0.0%
*Streptococcus sanguinis*	6.3%	3.2%

**Table 4:** Distribution of infectious agents according to onset.

	Early-Onset	Late-Onset
	%	%
*Enterobacter cloacae*	0.0%	2%
*Enterococcus faecalis*	**32.2%**	**24%**
*Escherichia coli*	0.0%	12%
*Klebsiella pneumoniae*	0.0%	2%
*Staphylococcus aureus*	**57.1%**	**28%**
*Staphylococcus epidermidis*	0.0%	10%
*Streptococcus gallolyticus*	0.0%	2%
*Streptococcus mitis*	0.0%	2%
*Streptococcus pseudoporcinus*	0.0%	2%
*Streptococcus sanguinis*	**10.7%**	16%

**Table 5:** Distribution of infectious agent according to the time of onset and patient gender.

	Early-Onset		Late-Onset	
	Male	Female	Male	Female
*Enterobacter cloacae*	3.5%	0%	0%	0%
*Enterococcus faecalis*	17.8%	7.1	16	12
*Escherichia coli*	3.5	7.1	0%	6%
*Klebsiella pneumoniae*	0%	0%	2%	0%
*Staphylococcus aureus*	25	17.9	20%	16%
*Staphylococcus epidermidis*	0%	0%	6%	4%
*Streptococcus gallolyticus*	3.5	0%	0%	0%
*Streptococcus mitis*	7.1	3.5	8%	2%
*Streptococcus pseudoporcinus*	3.5	0%	0%	0%
*Streptococcus sanguinis*	0%	0%	6%	2%

**Table 6:** Distribution of secondary diagnoses according to age category in patients with prosthetic valve IE.

	Age			
	21–40	41–60	61–80	81+
Mitral valve insufficiency	3.4%	14.7%	73.2%	8.7%
Aortic valve insufficiency	3.1%	25.4%	65.2%	6.3%
Congestive heart failure	0.0%	22.6%	70.2%	7.2%
Essential hypertension	0.0%	13.7%	78.1%	8.2%
Urinary tract infection	3.2%	30.3%	64.4%	2.1%
Atrial fibrillation	0.0%	21.5%	72.4%	6.1%
Gastroesophageal reflux disease with esophagitis	15.6%	26.4%	46,5%	11.5%
Esophageal varices	0.0%	66.6%	33.4%	0.0%

**Table 7:** The distribution of secondary diagnoses based on patient gender.

	Male	Female
Mitral valve insufficiency	71.2%	52.1%
Aortic valve insufficiency	63.1%	35.7%
Congestive heart failure	61.4%	52.6%
Essential hypertension	89.1%	78.4%
Atrial fibrillation	21.3%	6.8%
Gastroesophageal reflux disease with esophagitis	29.2%	15.1%
Urinary tract infection	20.2%	47.1%
Esophageal varices	12.4%	0%

## Data Availability

The data presented in this study are available upon request. The data are not publicly available because of the confidentiality of health data.
